# 1′,1′′-Dimethyl-4′-phenyl­dispiro­[11*H*-indeno­[1,2-*b*]quinoxaline-11,2′-pyrrolidine-3′,3′′-piperidin]-4′′-one

**DOI:** 10.1107/S160053681302223X

**Published:** 2013-08-14

**Authors:** J. Suresh, R. A. Nagalakshmi, K. Malathi, R. R. Kumar, P. L. N. Lakshman

**Affiliations:** a Department of Physics, The Madura College, Madurai 625 011, India; b Department of Organic Chemistry, School of Chemistry, Madurai Kamaraj University, Madurai 625 021, India; c Department of Food Science and Technology, University of Ruhuna, Mapalana, Kamburupitiya 81100, Sri Lanka

## Abstract

In the title compound, C_30_H_28_N_4_O, the central pyrrolidine ring adopts an envelope conformation with the CH_2_ C atom as the flap. The quinoxaline and indene ring systems are planar, with r.m.s. deviations of 0.0165 and 0.0181 Å, respectively. The pyrrolidine ring mean plane forms dihedral angles of 88.84 (1) and 86.14 (1)° with the quinoxaline and indene ring systems, respectively. A weak intra­molecular C—H⋯N inter­action is observed. In the crystal, C—H⋯O inter­actions lead to helical supra­molecular chains along the *b* axis having a *C*(9) motif.

## Related literature

For the importance of spiro compounds, see: Kobayashi *et al.* (1991 ▶); James *et al.* (1991 ▶). For the importance of pyrrolidine derivatives, see: Amal Raj *et al.* (2003 ▶). For conformation analysis, see: Cremer & Pople (1975 ▶). For a related structure, see: Selvanayagam *et al.* (2011 ▶). For hydrogen-bond motifs, see: Bernstein *et al.* (1995 ▶).
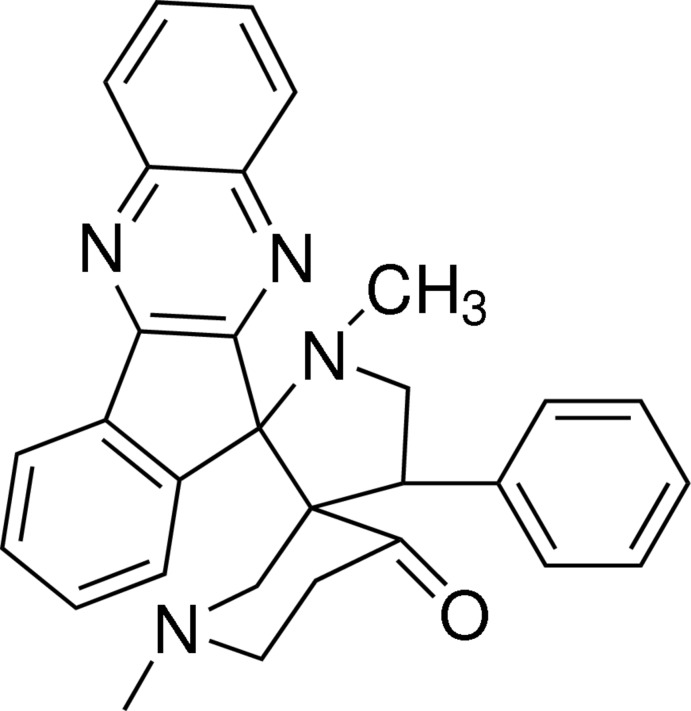


## Experimental

### 

#### Crystal data

C_30_H_28_N_4_O
*M* *_r_* = 460.56
Monoclinic, 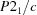

*a* = 13.4470 (6) Å
*b* = 8.4557 (4) Å
*c* = 20.8580 (9) Å
β = 90.195 (2)°
*V* = 2371.62 (19) Å^3^
*Z* = 4
Mo *K*α radiation
μ = 0.08 mm^−1^
*T* = 293 K
0.21 × 0.19 × 0.18 mm

#### Data collection

Bruker Kappa APEXII diffractometer
Absorption correction: multi-scan (*SADABS*; Sheldrick, 1996 ▶) *T*_min_ = 0.967, *T*_max_ = 0.974
28395 measured reflections
6662 independent reflections
4394 reflections with *I* > 2σ(*I*)
*R*_int_ = 0.032

#### Refinement

*R*[*F* ^2^ > 2σ(*F* ^2^)] = 0.050
*wR*(*F* ^2^) = 0.138
*S* = 1.02
6662 reflections
316 parameters
1 restraint
H-atom parameters constrained
Δρ_max_ = 0.19 e Å^−3^
Δρ_min_ = −0.23 e Å^−3^

### 

Data collection: *APEX2* (Bruker, 2004 ▶); cell refinement: *SAINT* (Bruker, 2004 ▶); data reduction: *SAINT*; program(s) used to solve structure: *SHELXS97* (Sheldrick, 2008 ▶); program(s) used to refine structure: *SHELXL97* (Sheldrick, 2008 ▶); molecular graphics: *PLATON* (Spek, 2009 ▶); software used to prepare material for publication: *SHELXL97*.

## Supplementary Material

Crystal structure: contains datablock(s) global, I. DOI: 10.1107/S160053681302223X/tk5246sup1.cif

Structure factors: contains datablock(s) I. DOI: 10.1107/S160053681302223X/tk5246Isup2.hkl

Additional supplementary materials:  crystallographic information; 3D view; checkCIF report

## Figures and Tables

**Table 1:** Hydrogen-bond geometry (Å, °)

*D*—H⋯*A*	*D*—H	H⋯*A*	*D*⋯*A*	*D*—H⋯*A*
C41—H41*B*⋯N3	0.97	2.39	2.9475 (18)	116
C9—H9⋯O1^i^	0.93	2.53	3.3564 (18)	148

Symmetry code: (i) 
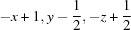
.

**Fig. 1.**
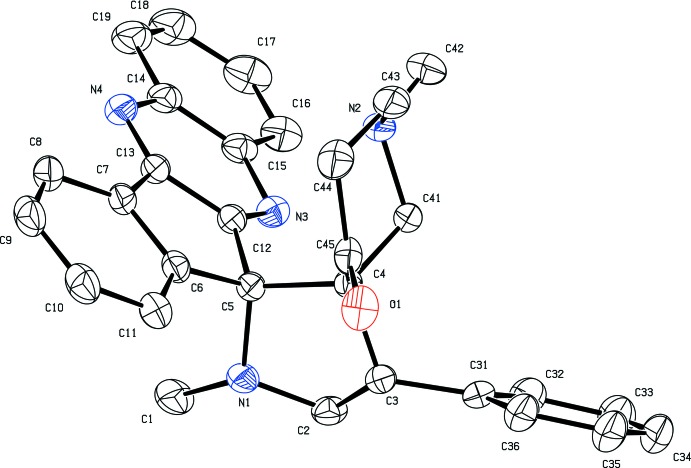
The molecular structure of (I), showing 20% probability displacement ellipsoids and the atom-numbering scheme. H-atoms are omitted for clarity.

**Fig. 2.**
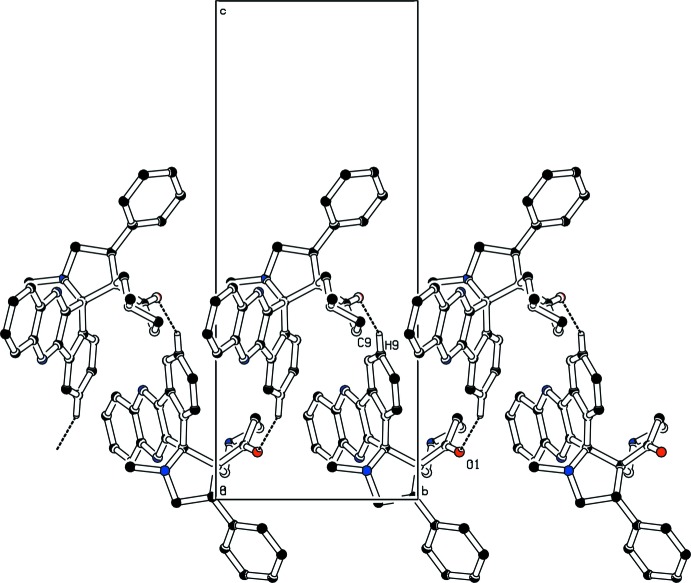
The partial packing diagram showing C—H—O interactions as dashed lines.

## supplementary crystallographic information

### 1. Comment

Spiro compounds are a particular class of naturally occurring substances characterized by highly pronounced biological properties (Kobayashi *et al.*, 1991; James *et al.*, 1991). Pyrrolidine derivatives are found to have anti-convulsant, anti-microbial and anti-fungal activities against various pathogens (Amal Raj *et al.*, 2003). Our interest in preparing pharmacologically active pyrrolidines led us to the title compound, and we have undertaken X-ray crystal structure determination in order to establish its conformation.

In the title compound (Fig. 1) C_30_H_28_N_4_O, the central pyrrolidine ring is an envelope on C2 with the asymmetry parameters ΔC_s_(C2) = 1.02 (13)° and puckering parameters q_2_ = 0.4394 (14) Å and φ_2_ = 219.24 (18)° (Cremer & Pople, 1975). The quinoxaline indene and the indole group forms dihedral angles of 88.84 (1) and 86.14 (1)°, respectively, with the central pyrrolidine ring. The quinoxaline-indene ring system (C12-C17/N3,N4) is planar, with r.m.s. deviation = 0.0165 Å. The indole group is also in planar with r.m.s. deviation = 0.0181 Å . The substituent at C3 is in an equatorial position indicated by the dihedral angle of 77.13 (1)° with the mean plane of the central pyrrolidine ring. The C—C bond lengths in the pyrrolidine ring in particular, at two spiro junctions (C3—C4 = 1.5531 (17) Å and C4—C5 = 1.6003 (17) Å) are somewhat longer than the normal values (C—C = 1.54 Å ), as found in a similar structure (Selvanayagam *et al.*, 2011). This may be due to the the steric interactions of the bulky substituents at atoms C4 and C5 of the pyrrolidine ring. The short H32 ··· H2A contact (2.27 Å) results in substantial widening of the bond angle C3—C31—C32 to 123.14 (14)°. The sum of bond angles around N1 (341.8°) and N2 (339.7°) indicate the atoms N1 and N2 are each in a trigonal geometry. A weak intramolecular C—H···N interaction is observed (Table 1).

In the crystal structure, a C—H···O interaction leads to helical chains generating a C_2_^2^(9) motif (Bernstein *et al.*, 1995).

### 2. Experimental

A mixture of 1-methyl-3-[*E*-phenylmethylidene]tetrahydro-2(1*H*)-pyridinone (1 mmol), ninhydrin (1 mmol), *o*-phenylenediamine (1 mmol) and sarcosine (1 mmol) in methanol was refluxed for 3-4 h. After completion of the reaction, as indicated by TLC, the reaction mixture was poured into cold water. The solid precipitate obtained was filtered and dried. The product was purified by column chromatography using petroleum ether:ethylacetate mixture (90:*10 v*/*v*). Suitable crystals for the single crystal X-ray studies were obtained by recrystallizing the product from methanol. Yield: 45%, M.pt: 510-511 K.

### 3. Refinement

H atoms were placed at calculated positions and allowed to ride on their carrier atoms with C—H = 0.93–0.98Å. *U*_iso_ = 1.2*U*_eq_(C) for CH_2_ and CH groups and *U*_iso_ = 1.5*U*_eq_(C) for CH_3_ groups. The reflections (1 0 0) and (0 0 2) are affected by the beam-stop and these reflections are omitted from the final refinement. The DELU constraint is applied to the N2—C43 bond in order to avoid the Hirshfield difference.

### Crystal data

**Table:**

C_30_H_28_N_4_O	*F*(000) = 976
*M**_r_* = 460.56	*D*_x_ = 1.290 Mg m^−^^3^
Monoclinic, *P*2_1_/*c*	Mo *K*α radiation, λ = 0.71073 Å
Hall symbol: -P 2ybc	Cell parameters from 2000 reflections
*a* = 13.4470 (6) Å	θ = 2–31°
*b* = 8.4557 (4) Å	µ = 0.08 mm^−^^1^
*c* = 20.8580 (9) Å	*T* = 293 K
β = 90.195 (2)°	Block, green
*V* = 2371.62 (19) Å^3^	0.21 × 0.19 × 0.18 mm
*Z* = 4	

### Data collection

**Table:**

Bruker Kappa APEXII diffractometer	6662 independent reflections
Radiation source: fine-focus sealed tube	4394 reflections with *I* > 2σ(*I*)
Graphite monochromator	*R*_int_ = 0.032
Detector resolution: 0 pixels mm^-1^	θ_max_ = 29.7°, θ_min_ = 2.5°
ω and φ scans	*h* = −18→13
Absorption correction: multi-scan (*SADABS*; Sheldrick, 1996)	*k* = −11→11
*T*_min_ = 0.967, *T*_max_ = 0.974	*l* = −18→28
28395 measured reflections	

### Refinement

**Table:**

Refinement on *F*^2^	Primary atom site location: structure-invariant direct methods
Least-squares matrix: full	Secondary atom site location: difference Fourier map
*R*[*F*^2^ > 2σ(*F*^2^)] = 0.050	Hydrogen site location: inferred from neighbouring sites
*wR*(*F*^2^) = 0.138	H-atom parameters constrained
*S* = 1.02	*w* = 1/[σ^2^(*F*_o_^2^) + (0.0621*P*)^2^ + 0.385*P*] where *P* = (*F*_o_^2^ + 2*F*_c_^2^)/3
6662 reflections	(Δ/σ)_max_ < 0.001
316 parameters	Δρ_max_ = 0.19 e Å^−^^3^
1 restraint	Δρ_min_ = −0.23 e Å^−^^3^

### Special details

**Table:**

Geometry. All e.s.d.'s (except the e.s.d. in the dihedral angle between two l.s. planes) are estimated using the full covariance matrix. The cell e.s.d.'s are taken into account individually in the estimation of e.s.d.'s in distances, angles and torsion angles; correlations between e.s.d.'s in cell parameters are only used when they are defined by crystal symmetry. An approximate (isotropic) treatment of cell e.s.d.'s is used for estimating e.s.d.'s involving l.s. planes.
Refinement. Refinement of *F*^2^ against ALL reflections. The weighted *R*- factor *wR* and goodness of fit *S* are based on *F*^2^, conventional *R*-factors *R* are based on *F*, with *F* set to zero for negative *F*^2^. The threshold expression of *F*^2^ > 2sigma(*F*^2^) is used only for calculating *R*-factors (gt) etc. and is not relevant to the choice of reflections for refinement. *R*-factors based on *F*^2^ are statistically about twice as large as those based on *F*, and *R*- factors based on ALL data will be even larger.

### Fractional atomic coordinates and isotropic or equivalent isotropic displacement parameters (Å^2^)

**Table:**

	*x*	*y*	*z*	*U*_iso_*/*U*_eq_	
C1	0.36237 (14)	0.57618 (18)	0.06530 (9)	0.0608 (4)	
H1A	0.3764	0.5501	0.1092	0.091*	
H1B	0.4125	0.5310	0.0383	0.091*	
H1C	0.2985	0.5346	0.0535	0.091*	
C2	0.34240 (11)	0.80537 (17)	−0.00602 (7)	0.0444 (3)	
H2A	0.2771	0.7738	−0.0212	0.053*	
H2B	0.3924	0.7701	−0.0362	0.053*	
C3	0.34818 (10)	0.98182 (15)	0.00505 (6)	0.0367 (3)	
H3	0.4176	1.0048	0.0159	0.044*	
C4	0.28798 (9)	1.00430 (14)	0.06781 (6)	0.0329 (3)	
C5	0.30193 (10)	0.83818 (15)	0.10348 (6)	0.0355 (3)	
C6	0.35593 (10)	0.84200 (15)	0.16788 (6)	0.0393 (3)	
C7	0.30027 (11)	0.77358 (16)	0.21664 (6)	0.0408 (3)	
C8	0.33822 (13)	0.75968 (19)	0.27843 (7)	0.0529 (4)	
H8	0.3010	0.7129	0.3108	0.063*	
C9	0.43177 (13)	0.8165 (2)	0.29050 (8)	0.0597 (4)	
H9	0.4581	0.8091	0.3317	0.072*	
C10	0.48776 (13)	0.8847 (2)	0.24228 (8)	0.0584 (4)	
H10	0.5509	0.9232	0.2516	0.070*	
C11	0.45123 (11)	0.89637 (18)	0.18041 (8)	0.0491 (4)	
H11	0.4898	0.9398	0.1479	0.059*	
C12	0.20533 (10)	0.75594 (14)	0.12273 (6)	0.0363 (3)	
C13	0.20671 (10)	0.71782 (15)	0.18953 (6)	0.0392 (3)	
C14	0.05869 (11)	0.59550 (16)	0.17986 (7)	0.0458 (3)	
C15	0.05754 (11)	0.63118 (16)	0.11364 (7)	0.0431 (3)	
C16	−0.02353 (12)	0.58276 (19)	0.07619 (9)	0.0541 (4)	
H16	−0.0262	0.6090	0.0329	0.065*	
C17	−0.09862 (13)	0.4972 (2)	0.10312 (10)	0.0672 (5)	
H17	−0.1520	0.4645	0.0779	0.081*	
C18	−0.09591 (14)	0.4583 (2)	0.16794 (11)	0.0701 (5)	
H18	−0.1470	0.3981	0.1854	0.084*	
C19	−0.02015 (13)	0.5068 (2)	0.20580 (9)	0.0600 (4)	
H19	−0.0200	0.4815	0.2492	0.072*	
C31	0.32246 (11)	1.08677 (18)	−0.05113 (6)	0.0436 (3)	
C32	0.25500 (13)	1.0445 (2)	−0.09802 (7)	0.0615 (4)	
H32	0.2198	0.9503	−0.0943	0.074*	
C33	0.23919 (18)	1.1419 (3)	−0.15091 (9)	0.0826 (7)	
H33	0.1942	1.1119	−0.1826	0.099*	
C34	0.2896 (2)	1.2812 (3)	−0.15630 (9)	0.0873 (7)	
H34	0.2787	1.3462	−0.1916	0.105*	
C35	0.35562 (18)	1.3253 (3)	−0.11021 (10)	0.0787 (6)	
H35	0.3894	1.4209	−0.1138	0.094*	
C36	0.37261 (13)	1.2288 (2)	−0.05827 (8)	0.0594 (4)	
H36	0.4187	1.2594	−0.0273	0.071*	
C41	0.17892 (10)	1.04422 (16)	0.05398 (6)	0.0379 (3)	
H41A	0.1758	1.1387	0.0277	0.045*	
H41B	0.1490	0.9583	0.0299	0.045*	
C42	0.01616 (12)	1.0763 (2)	0.09830 (9)	0.0662 (5)	
H42A	−0.0039	0.9799	0.0777	0.099*	
H42B	0.0028	1.1641	0.0704	0.099*	
H42C	−0.0203	1.0891	0.1375	0.099*	
C43	0.15684 (14)	1.21341 (18)	0.14362 (8)	0.0576 (4)	
H43A	0.1172	1.2339	0.1815	0.069*	
H43B	0.1493	1.3024	0.1147	0.069*	
C44	0.26476 (14)	1.19575 (19)	0.16236 (7)	0.0571 (4)	
H44A	0.2894	1.2966	0.1779	0.069*	
H44B	0.2700	1.1201	0.1972	0.069*	
C45	0.32857 (11)	1.14165 (16)	0.10784 (6)	0.0419 (3)	
N1	0.36204 (9)	0.74661 (13)	0.05769 (6)	0.0422 (3)	
N2	0.12222 (9)	1.07001 (14)	0.11253 (5)	0.0457 (3)	
N3	0.13447 (9)	0.71125 (13)	0.08434 (5)	0.0408 (3)	
N4	0.13632 (9)	0.63976 (14)	0.21868 (6)	0.0469 (3)	
O1	0.40639 (9)	1.20595 (13)	0.09512 (5)	0.0567 (3)	

### Atomic displacement parameters (Å^2^)

**Table:**

	*U*^11^	*U*^22^	*U*^33^	*U*^12^	*U*^13^	*U*^23^
C1	0.0738 (11)	0.0356 (8)	0.0732 (11)	0.0120 (8)	0.0169 (9)	−0.0021 (7)
C2	0.0490 (8)	0.0435 (8)	0.0408 (7)	0.0026 (6)	0.0096 (6)	−0.0072 (6)
C3	0.0359 (7)	0.0398 (7)	0.0344 (6)	−0.0005 (5)	0.0027 (5)	−0.0005 (5)
C4	0.0381 (7)	0.0314 (6)	0.0292 (6)	0.0015 (5)	0.0004 (5)	−0.0012 (5)
C5	0.0388 (7)	0.0328 (6)	0.0348 (6)	0.0028 (5)	0.0014 (5)	0.0003 (5)
C6	0.0426 (7)	0.0342 (6)	0.0411 (7)	0.0081 (6)	−0.0034 (6)	0.0007 (6)
C7	0.0473 (8)	0.0370 (7)	0.0382 (7)	0.0111 (6)	−0.0002 (6)	0.0027 (6)
C8	0.0624 (10)	0.0552 (9)	0.0411 (8)	0.0167 (8)	−0.0013 (7)	0.0061 (7)
C9	0.0654 (11)	0.0668 (10)	0.0466 (9)	0.0209 (9)	−0.0182 (8)	−0.0010 (8)
C10	0.0520 (9)	0.0629 (10)	0.0604 (10)	0.0118 (8)	−0.0172 (8)	−0.0023 (8)
C11	0.0458 (8)	0.0481 (8)	0.0535 (9)	0.0051 (7)	−0.0059 (7)	0.0022 (7)
C12	0.0424 (7)	0.0292 (6)	0.0374 (7)	0.0040 (5)	0.0036 (5)	−0.0002 (5)
C13	0.0474 (8)	0.0326 (6)	0.0378 (7)	0.0073 (6)	0.0062 (6)	0.0019 (5)
C14	0.0480 (8)	0.0351 (7)	0.0545 (9)	0.0054 (6)	0.0139 (7)	0.0006 (6)
C15	0.0430 (8)	0.0329 (7)	0.0534 (8)	0.0008 (6)	0.0091 (6)	−0.0055 (6)
C16	0.0509 (9)	0.0466 (8)	0.0648 (10)	−0.0048 (7)	0.0039 (7)	−0.0112 (7)
C17	0.0481 (10)	0.0606 (11)	0.0930 (14)	−0.0111 (8)	0.0067 (9)	−0.0129 (10)
C18	0.0543 (10)	0.0594 (11)	0.0969 (15)	−0.0102 (8)	0.0240 (10)	0.0029 (10)
C19	0.0561 (10)	0.0533 (9)	0.0707 (11)	−0.0002 (8)	0.0227 (8)	0.0073 (8)
C31	0.0459 (8)	0.0520 (8)	0.0330 (7)	0.0083 (6)	0.0100 (6)	0.0015 (6)
C32	0.0652 (11)	0.0785 (12)	0.0406 (8)	0.0092 (9)	−0.0033 (7)	−0.0034 (8)
C33	0.0974 (16)	0.1114 (18)	0.0389 (9)	0.0342 (14)	−0.0113 (9)	−0.0023 (10)
C34	0.1221 (19)	0.0938 (17)	0.0461 (11)	0.0480 (15)	0.0199 (12)	0.0228 (11)
C35	0.1013 (16)	0.0702 (12)	0.0647 (12)	0.0143 (11)	0.0216 (11)	0.0275 (10)
C36	0.0686 (11)	0.0565 (10)	0.0534 (9)	0.0014 (8)	0.0094 (8)	0.0130 (8)
C41	0.0420 (7)	0.0379 (7)	0.0338 (6)	0.0058 (6)	0.0033 (5)	0.0028 (5)
C42	0.0499 (10)	0.0749 (12)	0.0741 (11)	0.0191 (9)	0.0175 (8)	0.0087 (9)
C43	0.0811 (12)	0.0435 (8)	0.0485 (9)	0.0169 (7)	0.0164 (8)	−0.0054 (6)
C44	0.0888 (13)	0.0429 (8)	0.0396 (8)	0.0051 (8)	−0.0033 (8)	−0.0118 (6)
C45	0.0560 (9)	0.0333 (7)	0.0363 (7)	0.0012 (6)	−0.0102 (6)	0.0011 (5)
N1	0.0483 (7)	0.0334 (6)	0.0450 (6)	0.0073 (5)	0.0081 (5)	−0.0017 (5)
N2	0.0499 (7)	0.0447 (6)	0.0426 (6)	0.0114 (5)	0.0118 (5)	0.0014 (5)
N3	0.0451 (7)	0.0362 (6)	0.0410 (6)	−0.0024 (5)	0.0037 (5)	−0.0034 (5)
N4	0.0512 (7)	0.0441 (7)	0.0456 (7)	0.0052 (6)	0.0114 (6)	0.0063 (5)
O1	0.0632 (7)	0.0481 (6)	0.0586 (7)	−0.0154 (5)	−0.0148 (5)	0.0004 (5)

### Geometric parameters (Å, º)

**Table:**

C1—N1	1.4499 (18)	C15—C16	1.400 (2)
C1—H1A	0.9600	C16—C17	1.365 (2)
C1—H1B	0.9600	C16—H16	0.9300
C1—H1C	0.9600	C17—C18	1.392 (3)
C2—N1	1.4423 (18)	C17—H17	0.9300
C2—C3	1.5117 (19)	C18—C19	1.351 (3)
C2—H2A	0.9700	C18—H18	0.9300
C2—H2B	0.9700	C19—H19	0.9300
C3—C31	1.5091 (18)	C31—C32	1.379 (2)
C3—C4	1.5531 (17)	C31—C36	1.385 (2)
C3—H3	0.9800	C32—C33	1.393 (3)
C4—C45	1.5301 (18)	C32—H32	0.9300
C4—C41	1.5312 (18)	C33—C34	1.364 (3)
C4—C5	1.6003 (17)	C33—H33	0.9300
C5—N1	1.4731 (17)	C34—C35	1.359 (3)
C5—C6	1.5252 (18)	C34—H34	0.9300
C5—C12	1.5284 (19)	C35—C36	1.375 (2)
C6—C11	1.386 (2)	C35—H35	0.9300
C6—C7	1.3908 (19)	C36—H36	0.9300
C7—C8	1.389 (2)	C41—N2	1.4582 (17)
C7—C13	1.456 (2)	C41—H41A	0.9700
C8—C9	1.369 (2)	C41—H41B	0.9700
C8—H8	0.9300	C42—N2	1.457 (2)
C9—C10	1.384 (3)	C42—H42A	0.9600
C9—H9	0.9300	C42—H42B	0.9600
C10—C11	1.383 (2)	C42—H42C	0.9600
C10—H10	0.9300	C43—N2	1.451 (2)
C11—H11	0.9300	C43—C44	1.509 (2)
C12—N3	1.2986 (17)	C43—H43A	0.9700
C12—C13	1.4302 (19)	C43—H43B	0.9700
C13—N4	1.3058 (18)	C44—C45	1.498 (2)
C14—N4	1.371 (2)	C44—H44A	0.9700
C14—C19	1.408 (2)	C44—H44B	0.9700
C14—C15	1.414 (2)	C45—O1	1.2095 (18)
C15—N3	1.3807 (18)		
			
N1—C1—H1A	109.5	C16—C17—C18	120.53 (17)
N1—C1—H1B	109.5	C16—C17—H17	119.7
H1A—C1—H1B	109.5	C18—C17—H17	119.7
N1—C1—H1C	109.5	C19—C18—C17	120.90 (17)
H1A—C1—H1C	109.5	C19—C18—H18	119.5
H1B—C1—H1C	109.5	C17—C18—H18	119.5
N1—C2—C3	100.96 (11)	C18—C19—C14	120.29 (17)
N1—C2—H2A	111.6	C18—C19—H19	119.9
C3—C2—H2A	111.6	C14—C19—H19	119.9
N1—C2—H2B	111.6	C32—C31—C36	117.89 (15)
C3—C2—H2B	111.6	C32—C31—C3	123.14 (14)
H2A—C2—H2B	109.4	C36—C31—C3	118.87 (13)
C31—C3—C2	116.76 (11)	C31—C32—C33	120.5 (2)
C31—C3—C4	117.63 (11)	C31—C32—H32	119.8
C2—C3—C4	102.87 (10)	C33—C32—H32	119.8
C31—C3—H3	106.2	C34—C33—C32	120.1 (2)
C2—C3—H3	106.2	C34—C33—H33	119.9
C4—C3—H3	106.2	C32—C33—H33	119.9
C45—C4—C41	105.97 (10)	C35—C34—C33	120.16 (18)
C45—C4—C3	111.52 (11)	C35—C34—H34	119.9
C41—C4—C3	111.71 (10)	C33—C34—H34	119.9
C45—C4—C5	111.80 (10)	C34—C35—C36	120.1 (2)
C41—C4—C5	113.06 (10)	C34—C35—H35	120.0
C3—C4—C5	102.93 (9)	C36—C35—H35	120.0
N1—C5—C6	108.76 (10)	C35—C36—C31	121.31 (19)
N1—C5—C12	113.57 (11)	C35—C36—H36	119.3
C6—C5—C12	100.41 (10)	C31—C36—H36	119.3
N1—C5—C4	102.92 (10)	N2—C41—C4	112.24 (10)
C6—C5—C4	116.47 (10)	N2—C41—H41A	109.2
C12—C5—C4	115.03 (10)	C4—C41—H41A	109.2
C11—C6—C7	119.99 (13)	N2—C41—H41B	109.2
C11—C6—C5	127.61 (13)	C4—C41—H41B	109.2
C7—C6—C5	112.30 (12)	H41A—C41—H41B	107.9
C8—C7—C6	121.10 (14)	N2—C42—H42A	109.5
C8—C7—C13	130.31 (14)	N2—C42—H42B	109.5
C6—C7—C13	108.49 (11)	H42A—C42—H42B	109.5
C9—C8—C7	118.38 (16)	N2—C42—H42C	109.5
C9—C8—H8	120.8	H42A—C42—H42C	109.5
C7—C8—H8	120.8	H42B—C42—H42C	109.5
C8—C9—C10	120.94 (14)	N2—C43—C44	109.87 (12)
C8—C9—H9	119.5	N2—C43—H43A	109.7
C10—C9—H9	119.5	C44—C43—H43A	109.7
C11—C10—C9	121.03 (16)	N2—C43—H43B	109.7
C11—C10—H10	119.5	C44—C43—H43B	109.7
C9—C10—H10	119.5	H43A—C43—H43B	108.2
C10—C11—C6	118.53 (15)	C45—C44—C43	112.72 (12)
C10—C11—H11	120.7	C45—C44—H44A	109.0
C6—C11—H11	120.7	C43—C44—H44A	109.0
N3—C12—C13	122.84 (13)	C45—C44—H44B	109.0
N3—C12—C5	126.36 (12)	C43—C44—H44B	109.0
C13—C12—C5	110.50 (11)	H44A—C44—H44B	107.8
N4—C13—C12	124.08 (13)	O1—C45—C44	121.83 (13)
N4—C13—C7	127.56 (13)	O1—C45—C4	121.92 (13)
C12—C13—C7	108.29 (12)	C44—C45—C4	116.22 (13)
N4—C14—C19	119.42 (15)	C2—N1—C1	116.35 (12)
N4—C14—C15	121.63 (13)	C2—N1—C5	108.49 (10)
C19—C14—C15	118.90 (15)	C1—N1—C5	116.94 (12)
N3—C15—C16	118.66 (14)	C43—N2—C42	111.90 (13)
N3—C15—C14	122.10 (13)	C43—N2—C41	109.35 (12)
C16—C15—C14	119.24 (14)	C42—N2—C41	110.44 (12)
C17—C16—C15	120.08 (17)	C12—N3—C15	114.77 (12)
C17—C16—H16	120.0	C13—N4—C14	114.51 (12)
C15—C16—H16	120.0		
			
N1—C2—C3—C31	−174.91 (11)	N3—C15—C16—C17	177.03 (14)
N1—C2—C3—C4	−44.52 (13)	C14—C15—C16—C17	−2.4 (2)
C31—C3—C4—C45	−82.17 (14)	C15—C16—C17—C18	0.5 (3)
C2—C3—C4—C45	147.97 (11)	C16—C17—C18—C19	1.3 (3)
C31—C3—C4—C41	36.21 (16)	C17—C18—C19—C14	−1.2 (3)
C2—C3—C4—C41	−93.65 (12)	N4—C14—C19—C18	−178.14 (15)
C31—C3—C4—C5	157.82 (11)	C15—C14—C19—C18	−0.7 (2)
C2—C3—C4—C5	27.95 (12)	C2—C3—C31—C32	30.6 (2)
C45—C4—C5—N1	−121.60 (11)	C4—C3—C31—C32	−92.51 (16)
C41—C4—C5—N1	118.90 (11)	C2—C3—C31—C36	−145.75 (14)
C3—C4—C5—N1	−1.78 (12)	C4—C3—C31—C36	91.18 (16)
C45—C4—C5—C6	−2.74 (15)	C36—C31—C32—C33	0.6 (2)
C41—C4—C5—C6	−122.24 (12)	C3—C31—C32—C33	−175.77 (15)
C3—C4—C5—C6	117.08 (12)	C31—C32—C33—C34	−0.9 (3)
C45—C4—C5—C12	114.35 (13)	C32—C33—C34—C35	0.2 (3)
C41—C4—C5—C12	−5.15 (14)	C33—C34—C35—C36	0.6 (3)
C3—C4—C5—C12	−125.83 (11)	C34—C35—C36—C31	−0.9 (3)
N1—C5—C6—C11	56.97 (17)	C32—C31—C36—C35	0.3 (2)
C12—C5—C6—C11	176.43 (13)	C3—C31—C36—C35	176.80 (15)
C4—C5—C6—C11	−58.68 (18)	C45—C4—C41—N2	−56.74 (14)
N1—C5—C6—C7	−119.49 (12)	C3—C4—C41—N2	−178.38 (11)
C12—C5—C6—C7	−0.03 (13)	C5—C4—C41—N2	66.07 (13)
C4—C5—C6—C7	124.86 (12)	N2—C43—C44—C45	51.34 (18)
C11—C6—C7—C8	0.6 (2)	C43—C44—C45—O1	132.24 (15)
C5—C6—C7—C8	177.34 (12)	C43—C44—C45—C4	−45.70 (17)
C11—C6—C7—C13	−176.19 (12)	C41—C4—C45—O1	−131.69 (13)
C5—C6—C7—C13	0.57 (15)	C3—C4—C45—O1	−9.93 (17)
C6—C7—C8—C9	0.6 (2)	C5—C4—C45—O1	104.71 (14)
C13—C7—C8—C9	176.60 (14)	C41—C4—C45—C44	46.25 (14)
C7—C8—C9—C10	−0.7 (2)	C3—C4—C45—C44	168.01 (11)
C8—C9—C10—C11	−0.5 (3)	C5—C4—C45—C44	−77.35 (14)
C9—C10—C11—C6	1.7 (2)	C3—C2—N1—C1	−179.96 (13)
C7—C6—C11—C10	−1.7 (2)	C3—C2—N1—C5	45.72 (14)
C5—C6—C11—C10	−177.93 (13)	C6—C5—N1—C2	−151.26 (11)
N1—C5—C12—N3	−58.40 (17)	C12—C5—N1—C2	97.86 (13)
C6—C5—C12—N3	−174.31 (12)	C4—C5—N1—C2	−27.15 (13)
C4—C5—C12—N3	59.83 (17)	C6—C5—N1—C1	74.73 (16)
N1—C5—C12—C13	115.36 (12)	C12—C5—N1—C1	−36.15 (17)
C6—C5—C12—C13	−0.54 (13)	C4—C5—N1—C1	−161.16 (13)
C4—C5—C12—C13	−126.41 (11)	C44—C43—N2—C42	175.24 (13)
N3—C12—C13—N4	−2.3 (2)	C44—C43—N2—C41	−62.07 (16)
C5—C12—C13—N4	−176.31 (12)	C4—C41—N2—C43	67.73 (14)
N3—C12—C13—C7	174.93 (12)	C4—C41—N2—C42	−168.72 (12)
C5—C12—C13—C7	0.91 (14)	C13—C12—N3—C15	3.36 (18)
C8—C7—C13—N4	−0.2 (2)	C5—C12—N3—C15	176.41 (12)
C6—C7—C13—N4	176.18 (13)	C16—C15—N3—C12	178.09 (13)
C8—C7—C13—C12	−177.28 (14)	C14—C15—N3—C12	−2.53 (19)
C6—C7—C13—C12	−0.91 (15)	C12—C13—N4—C14	−0.02 (19)
N4—C14—C15—N3	0.5 (2)	C7—C13—N4—C14	−176.69 (13)
C19—C14—C15—N3	−176.95 (13)	C19—C14—N4—C13	178.22 (13)
N4—C14—C15—C16	179.83 (13)	C15—C14—N4—C13	0.82 (19)
C19—C14—C15—C16	2.4 (2)		

### Hydrogen-bond geometry (Å, º)

**Table:**

*D*—H···*A*	*D*—H	H···*A*	*D*···*A*	*D*—H···*A*
C41—H41*B*···N3	0.97	2.39	2.9475 (18)	116
C9—H9···O1^i^	0.93	2.53	3.3564 (18)	148

Symmetry code: (i) −*x*+1, *y*−1/2, −*z*+1/2.
